# Endovascular treatment of postoperative hemorrhage after pancreatectomy: a retrospective study

**DOI:** 10.1186/s12876-023-03022-9

**Published:** 2023-11-07

**Authors:** Hideki Izumi, Hisamichi Yoshii, Rika Fujino, Shigeya Takeo, Eiji Nomura, Masaya Mukai, Satoshi Suda, Kosuke Tomita, Shunsuke Kamei, Yukihisa Ogawa, Terumitsu Hasebe, Hiroyasu Makuuchi

**Affiliations:** 1 Department of Gastrointestinal Surgery, Tokai University Hachioji Hospital, Ishikawa, Hachioji, Tokyo, 1838, 192-0032 Japan; 2 Department of Radiology, Tokai University Hachioji Hospital, 1838 Ishikawa, Hachioji, Tokyo, 192-0032 Japan

**Keywords:** Gastroduodenal artery bleeding, Pseudoaneurysm, Pancreaticoduodenectomy, Viabahn stent graft

## Abstract

**Background:**

Ruptured aneurysm is a serious complication of distal pancreatectomy (DP) or pancreatoduodenectomy (PD) that can be life-threatening if not treated promptly. This study aimed to examine the efficacy of a Viabahn stent graft for stopping bleeding after pancreatectomy.

**Methods:**

Between April 2016 and June 2022, we performed 245 pancreatectomies in our institution. Six patients experienced postoperative bleeding and underwent endovascular treatment.

**Results:**

All six cases of bleeding occurred post-PD (3.7%). The bleeding was from gastroduodenal artery (GDA) pseudoaneurysms in three patients, and Viabahn stent grafts were inserted. All three patients did not show liver function abnormalities or hepatic blood flow disorders. One patient with a Viabahn stent graft experienced rebleeding, which required further management to obtain hemostasis. Of the six cases in which there was hemorrhage, one case of bleeding from the native hepatic artery could not be managed.

**Conclusions:**

Using the Viabahn stent graft is an effective treatment option for postoperative bleeding from GDA pseudoaneurysms following PD. In most cases, using this device resulted in successful hemostasis, without observed abnormalities in hepatic function or blood flow.

**Supplementary Information:**

The online version contains supplementary material available at 10.1186/s12876-023-03022-9.

## Background

Rupture of an aneurysm is a serious complication following distal pancreatectomy (DP) or pancreatoduodenectomy (PD) that can be life-threatening if not treated promptly [1, 2]. Post-pancreatectomy hemorrhage was reported in 3–16% of patients, with a mortality rate of 10–20% [2–5]. When blood vessels are exposed to high levels of amylase due to pancreatic leakage, the vessel wall becomes fragile, causing aneurysms [6, 7]. Transcatheter arterial embolization (TAE) can combat the bleeding, but blood flow impairment in eliminated organs and tissues is inevitable [8]. However, recent advances in vascular treatment have reported the effectiveness of the Viabahn stent graft, which allows hemostasis without impairing peripheral blood flow [9–11].

In December 2016, the Viabahn stent graft was adopted as the world’s first stent graft for injuries in Japan. The Viabahn stent graft is heparin-coated and self-expanding, combining high flexibility with delivery systems, and is considered effective in treating hemorrhage from pancreatectomy [10, 11]; however, there are also reports of complications [12].

In this study, we aimed to evaluate the management of bleeding after pancreatectomy and confirm the safety of Viabahn stent grafts.

## Methods

### Patients

A total of 245 pancreatectomies were performed between April 2016 and June 2022. PD was performed in 163 patients, DP in 75, and total pancreatectomy (TP) in 7. Bleeding occurred in six patients (2.4%). Dynamic contrast-enhanced computed tomography was performed in patients with suspected hemorrhage to confirm contrast leakage and aneurysm formation. Patient characteristics are summarized in Table S1.

This study conformed to the ethical principles of the Declaration of Helsinki, with approval from the institutional review board. Written informed consent was obtained from the patients for this study.

### Surgical procedures

#### Pancreaticoduodenectomy

The surgical procedure performed for all patients was subtotal stomach-preserving PD (SSPPD) via the modified Child’s method [13]. Pancreaticojejunal mucosal anastomosis was performed. No pancreatic duct stent was placed in patients with main pancreatic duct dilatation. For patients with a soft pancreas without main pancreatic duct dilation, a 5-Fr pancreatic duct stent (Sumitomo Bakelite, Tokyo, Japan) was placed in the jejunum as a lost stent. No external stent was used for pancreatoenterostomy. No external bile fistulas were found, and jejunostomy or gastrointestinal tube was not required. No measures, such as wrapping the gastroduodenal artery transection with the great omentum, were taken. Only one patient required the placement of a closed suction drain from the left side to the dorsal pancreaticojejunal anastomosis. The drain was checked for amylase on the third postoperative day and removed when there were no complications. The patients were discharged on postoperative day 7, when no postoperative problems developed.

#### Distal pancreatectomy

Whether laparoscopic or open, pancreatic dissection was performed using a Powered ECHELON FLEX® (Johnson & Johnson, Tokyo, Japan) 60-mm Green cartridge. No additional treatment was performed for vascular transection during DP or PD. A closed suction drain was placed through the patient’s right abdomen, and the patient underwent pancreatic dissection. The drain was checked for amylase on postoperative day 3 as well as on PD, and the drain was removed if no problems occurred.

### Techniques for Viabahn stent-graft placement

The decision to perform coil embolization versus place a Viabahn stent graft was at the physician’s discretion, based on the size of the vessel to be treated, length of the abnormality, adjacent arterial anatomy, and potential for distal organ ischemia. Endovascular treatment was performed by a physician with > 10 years of experience. The access route was selected from either the brachiocephalic or inguinal region according to the geometry of the celiac artery, referring to the contrast CT. A 6 F guiding sheath, 45 or 90 cm (Destination, Terumo, Tokyo, Japan), was inserted. The celiac artery was selected using a 4 F catheter (Cobra or Shepherd hook; Medikit, Tokyo, Japan), and a 0.035-inch radifocus guidewire was inserted into the right hepatic artery, A8. After replacing the catheter with an inserter attached to the sheath, the guiding sheath was inserted into the proper hepatic artery (PHA) or common hepatic artery (CHA). The diameter and length of the Viabahn stent graft were determined using quantitative vessel angiography (QVA) and preoperative contrast CT. After the wire was replaced with a micro guidewire V18 (Boston Scientific, Marlborough, MA) or Treasure floppy (Asahi Intecc, Aichi, Japan), the stent graft was inserted into the treatment site and positioned to provide adequate coverage of the gastroduodenal artery (GDA) transection. After the stent graft was inserted into the treatment site, the guiding sheath was pulled back sufficiently against the graft, and deployment was performed under fluoroscopic observation. Stent graft dilation was performed using a balloon catheter (Sterling, Boston Scientific, Marlborough, Massachusetts, MA, and Admiral Xtreme, Medtronic, Medtronic, Minneapolis, MN) of the corresponding diameter. The procedure was terminated after confirmation of stent patency and absence of an endoleak by contrast.

### Follow-up after vascular procedures

After intravascular hemostasis, patients were admitted to the intensive care unit, and blood samples were collected immediately after admission and the next morning. Three days after intravascular hemostasis, an arterial phase CT was performed to check for aneurysms and evaluate the patency of the Viabahn stent graft.

### Definitions and data collection

Data regarding age, sex, pathology name, personal history, surgical procedure, complications, information on bleeding (onset, site, and treatment), presence of rebleeding, emergency operation, and outcomes were collected from medical records. Postoperative complications were evaluated according to the modified Clavien–Dindo classification [14]. Adverse events were assessed according to the Common Terminology Criteria for Adverse Events (CTCAE) version 5.0, established by the National Cancer Institute. The data gathered were recorded in MS Excel.

## Results

The six cases of bleeding after pancreatectomy are summarized in Table S1. All patients underwent PD; no bleeding was observed in the DP and TP groups. Bleeding was observed in 6 of 163 patients with PD, resulting in a post-PD bleeding rate of 3.7%. All cases were post-PD: two cases of cholangiocarcinoma, two cases of duodenal gastrointestinal stromal tumor (GIST), and one case each of duodenal and pancreatic cancers. The mean age was 73.7 years (59–85 years), with 3 male and 3 female patients each. Two patients underwent SSPPD, and four underwent laparoscopic PD (Lap-PD).

The most common causes of bleeding were biliary leakage in four cases and pancreatic leakage in two cases. One patient had neither pancreatic nor biliary leakages. The average bleeding duration was 13.9 days (8–28 days) postoperatively, and signs of bleeding included bleeding from the drain in three cases and melena, anemia, and hematemesis in one case each.

Bleeding sites were GDA pseudoaneurysm in three cases (patients 1–3), dorsal pancreatic artery (DPA; patient 4), proper hepatic artery (patient 5), and right hepatic artery (patient 6). A Viabahn stent graft was inserted to stop bleeding from the GDA pseudoaneurysm in all three cases, whereas coil embolization was performed to stop bleeding from the DPA, proper hepatic artery, and right hepatic artery.

### Viabahn stent-graft

**Fig. 1:**
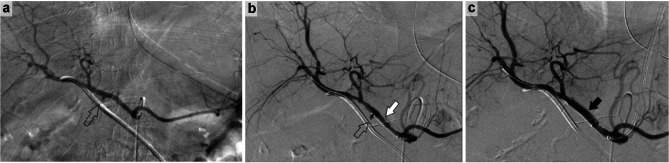
A 75-year-old woman with duodenal cancer experienced bleeding on postoperative day 14. **a**: Pseudoaneurysm at the GDA transection (⇧). **b**: The pseudoaneurysm at the GDA transection was located (⇧), and a Viabahn stent graft was placed in the appropriate position (white arrow). **c**: Viabahn stent graft was expanded, and hemostasis was completed (black arrow). *Abbreviation*: GDA, gastroduodenal artery

Patient 1 was a 75-year-old woman who underwent SSPPD for duodenal cancer. Postoperative pancreatic and bile leaks were observed and drained. Bleeding from the drain was observed on postoperative day 14. A GDA aneurysm was observed (Fig. 1a); hence, a Viabahn stent graft was delivered (Fig. 1b), and hemostasis was confirmed (Fig. 1c).

**Fig. 2:**
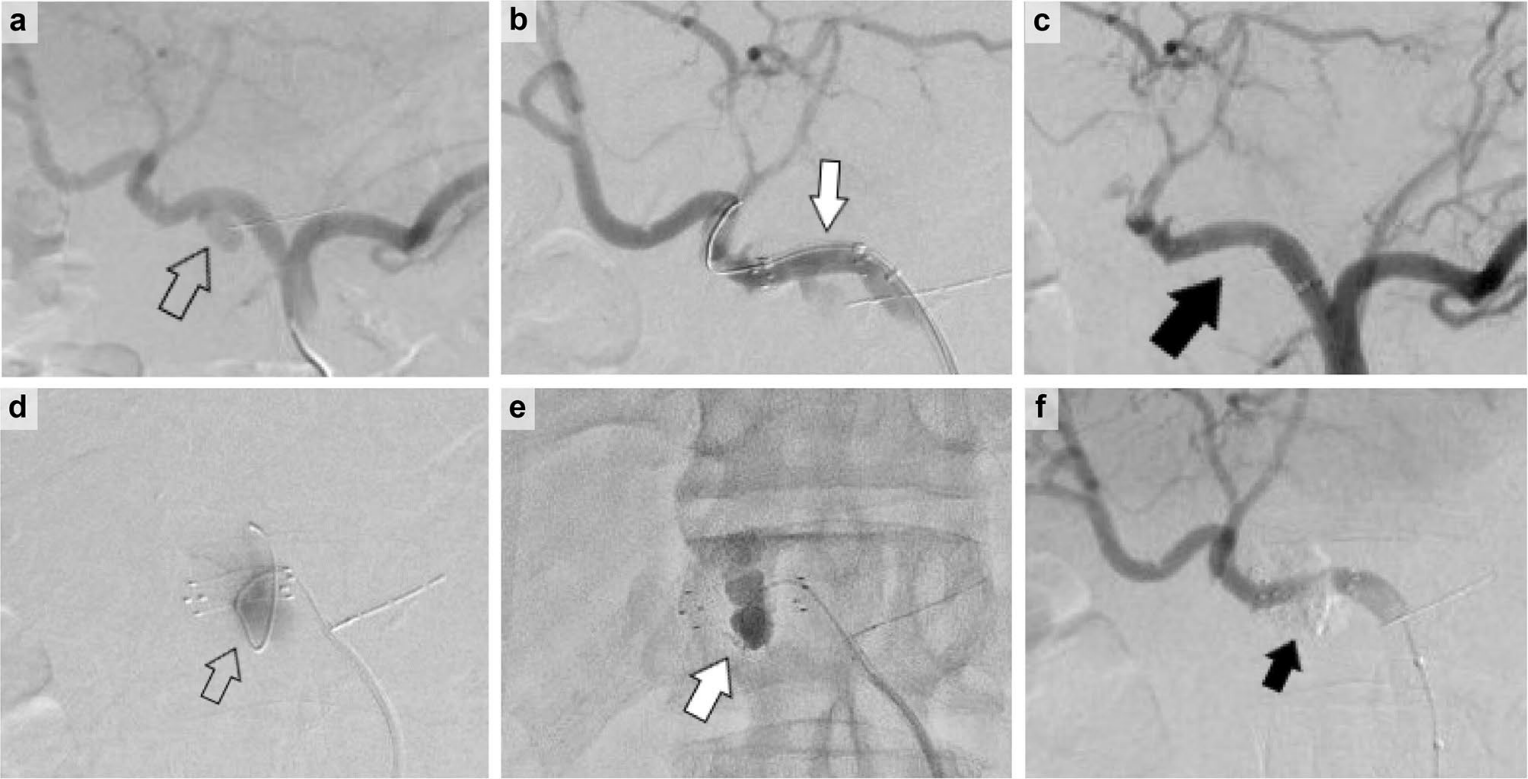
A 59-year-old male patient with cholangiocarcinoma on dialysis experiened bleeding on postoperative day 28. The patient had no evidence of pancreatic or biliary leakage. **a**: Pseudoaneurysm at the GDA transection (⇧). **b**: The GDA pseudoaneurysm was identified, and the position of the Viabahn stent graft was adjusted (white arrow). **c**: Viabahn stent graft was expanded, and hemostasis was completed (black arrow). The next day, the patient was found to be hypotensive and hemorrhagic, and another angiogram was performed because rebleeding was suspected on enhanced CT. **d**: The GDA pseudoaneurysm that had not been visualized the previous day was again contrasted, and a guidewire strayed into the pseudoaneurysm from the side of the Viabahn stent graft (⇧). **e**: The n-butyl-2-cyanoacrylate (NBCA) and lipiodol mixture was injected into the aneurysm, and the Viabahn stent graft was further balloon-dilated and compressed (white arrow). **f**: Hemostasis was achieved without compromising hepatic blood flow (black arrow). *Abbreviation*: GDA, gastroduodenal artery

Patient 2 was a 59-year-old man who underwent Lap-PD for cholangiocarcinoma. The patient had a good postoperative course and was discharged on postoperative day 15. On postoperative day 28, he was urgently brought to the hospital because of hemorrhagic shock. A GDA aneurysm was found (Fig. 2a), a Viabahn stent graft was inserted (Fig. 2b), and hemostasis was confirmed (Fig. 2c). However, the next day, the hemorrhage recurred; hence, re-hemostasis was performed. Angiography revealed that the guidewire entered the GDA aneurysm through the side of the Viabahn stent graft, and the contrast material entered the aneurysm (Fig. 2d). An n-buty1-2-cyanoacrylate (NBCA) and lipiodol mixture was injected into the aneurysm, and the Viabahn stent graft was recompressed with a balloon (Fig. 2e). Subsequently, complete hemostasis was confirmed (Fig. 2f).

**Fig. 3:**
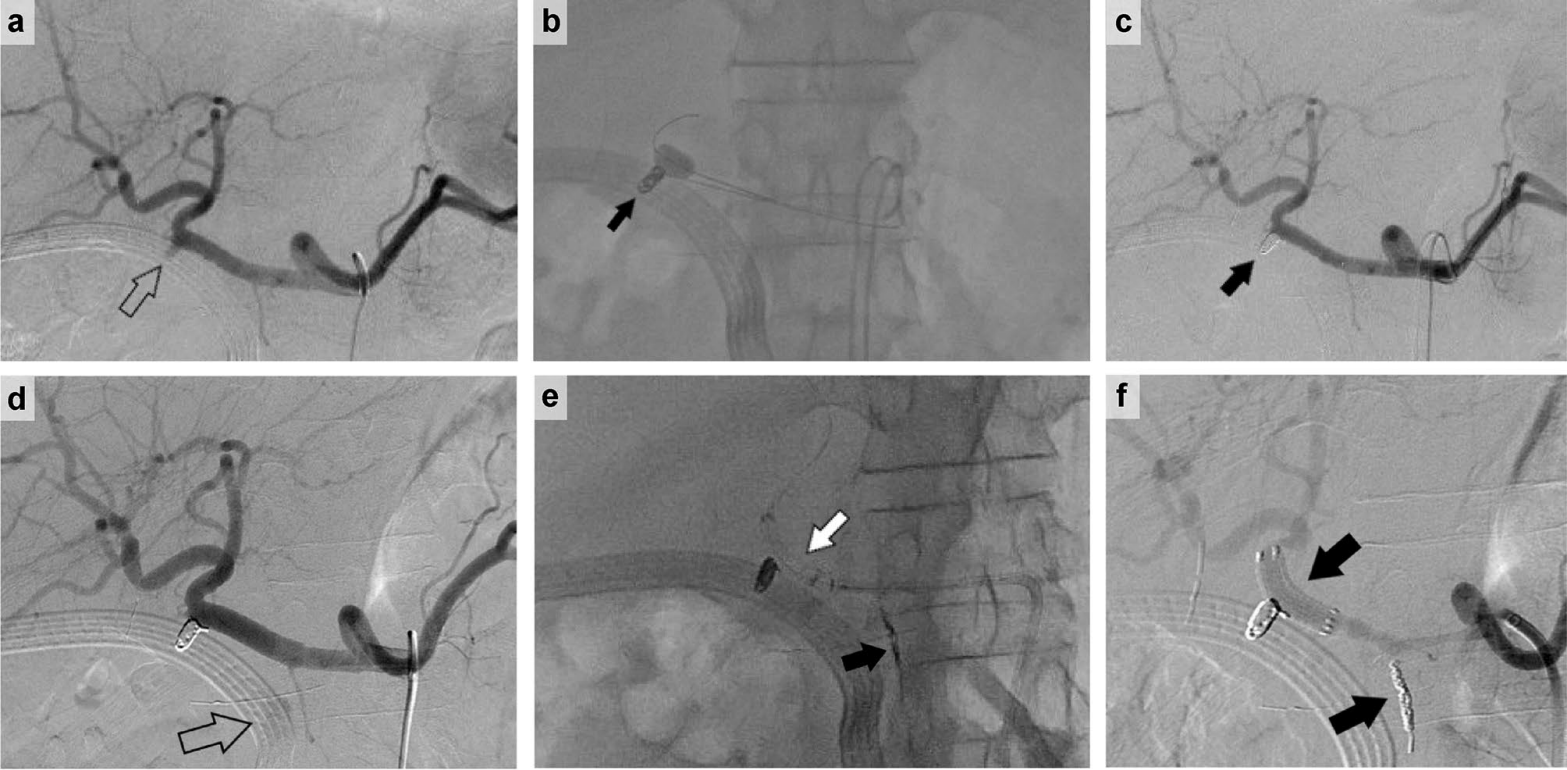
A 71-year-old man experienced bleeding on day 8 after duodenal GIST resection. **a**: Pseudoaneurysm of GDA (⇧). **b**: Since there was no evidence of active bleeding and the patient’s general condition was stable, the PHA was balloon-occluded, and hemostasis was achieved with coil embolization at the GDA transection (black arrow). **c**: Contrast image after GDA transection coil embolization (black arrow). The procedure was terminated after confirming that there was no bleeding and that the hepatic blood flow was preserved. The following day, bleeding from the drain was observed, and another angiogram was performed. **d**: Hemorrhage from the dorsal pancreatic artery was observed (⇧). **e**: The dorsal pancreatic artery is coil-embolized (black arrow), and a Viabahn stent graft was inserted to close the GDA coiling area (white arrow). **f**: The procedure was terminated after confirming hepatic arterial blood flow and hemostasis. *Abbreviations*: GDA, gastroduodenal artery; GIS, gastrointestinal stromal tumor; PHA, proper hepatic artery

Patient 3 was a 71-year-old man who was diagnosed with duodenal GIST and underwent Lap-PD. Postoperatively, the patient developed a bile leak, which was drained. On postoperative day 8, bleeding from the drain was observed. Although a GDA aneurysm was observed (Fig. 3a), the patient was not in shock, and there was no obvious contrast leakage; therefore, only GDA coiling was performed (Fig. 3b). No bleeding was observed immediately after the procedure (Fig. 3c). However, the following day, bleeding was observed again from the drain, coming from the DPA (Fig. 3d). Thus, the DPA was coiled to stop the bleeding, and a Viabahn stent graft was placed to close the GDA coiling site to avoid rebleeding (Fig. 3e). The procedure was terminated after confirming the absence of bleeding (Fig. 3f).

Rebleeding occurred in one case (patient 2) in which a Viabahn stent graft was inserted, the aneurysm was revascularized the next day, and coil embolization was performed in the aneurysm. None of the patients underwent reoperation. One patient (patient 5) who underwent coil embolization of the proper hepatic artery died without successful hemostasis; five cases, including three cases in which Viabahn stent grafts were inserted, resulted in successful hemostasis and were discharged without complications.

## Discussion

In this study, we report three cases of Viabahn stent graft insertion due to bleeding after pancreatectomy. None of the patients experienced bleeding after DP surgery, whereas all experienced bleeding after PD. The three patients who had a Viabahn stent graft inserted had aneurysms of the GDA. Insertion of the Viabahn stent grafts was safe and effective, and no complications, such as liver failure or liver abscess, were observed afterward.

The risk of bleeding after pancreatectomy is termed postoperative pancreatic fistula (POPF) [2, 15–17]. Wang et al. [15] have reported that in addition to POPF, older age and total bilirubin level were associated with bleeding risk after pancreatectomy. Vilhav et al. [18] have found that high C-reactive protein (CRP) levels at 5 or 6 days postoperatively are associated with bleeding. Interestingly, they reported no association between drain amylase values and hemorrhage, which could be because approximately half of the cases of hemorrhage were biochemical leaks. In our case, only two of the six patients with hemorrhage had Grade B pancreatic leakage. In contrast, four of the six cases had biliary leakage, suggesting that bile activation of pancreatic juice may cause aneurysms at the surgically operated GDA transects. Despite the small number of cases in the present study, the absence of bleeding after DP confirmed our findings.

The etiology of pseudoaneurysm formation has not been delineated. It is believed to be most commonly due to pancreatic fistula or anastomotic dehiscence [19, 20]. However, pseudoaneurysm can develop far from the pancreatic cut surface, and there is no evidence of pancreatic leak in some cases. It has been suggested that skeletonization of the visceral arteries may result in iatrogenic vascular injury (e.g., secondary to diathermy) [19, 21].

Post-pancreatectomy hemorrhage has been reported to occur in post-pancreatectomy patients [2–5]. In the past, vascular ligation by laparotomy was the mainstay of treatment for postoperative bleeding, but it is highly invasive and difficult to perform due to adhesions, with a low survival rate [22]. As expected, cases requiring intervention have a higher mortality rate than those that do not [1]. In recent years, transcatheter arterial embolization (TAE) was performed, but impaired blood flow in eliminated organs and tissues was inevitable [8]. Meta-analyses have reported mortality rates for endovascular treatment and reoperation. In a meta-analysis, the mortality rate was 43% for reoperation versus 21% for endovascular treatment, indicating the effectiveness of endovascular treatment [23]. Hepatic dysfunction after TAE of the common hepatic artery, especially to stop bleeding from gastroduodenal artery transection, occurs at a high rate of 30–50%, and liver abscesses occur in 10–13% of cases [24]. Most hemorrhage due to pancreatic leakage is from GDA stumps, and a Viabahn stent graft is excellent for stopping bleeding without impairing blood flow to the liver [25]. All three of our patients were discharged from the hospital without subsequent liver dysfunction or liver abscess.

The most common problem with the insertion of the Viabahn stent graft is rebleeding. The frequency of rebleeding in PD postoperative bleeding was 10–30% [26, 27]. The effectiveness of surgical reintervention decreases proportionally with the number of procedures; from 60% for the first reintervention to 50% for a second relaparotomy [15]. In one of our cases (patient 2), we encountered rebleeding from the side on postoperative day 2 after inserting a Viabahn stent graft. The other case (patient 3) had rebleeding from the DPA the day after GDA coiling. Although there was no bleeding from the GDA coiling site, the patient had rebleeding; hence, a prophylactic Viabahn stent graft was inserted to prevent further bleeding complications. Coiling of the GDA dissection is often associated with rebleeding [25, 28], and covered stent insertion seems to be the preferred method. For these reasons, an additional Viabahn stent graft was inserted in patient 3 because of the possibility of rebleeding.

Another problem with Viabahn stent grafts is occlusion. The reported patency rate of the Viabahn at 1 year varies from 40 to 80% [29–32]. The small stent diameter is a risk for thrombotic occlusion [29]. In contrast, there are reports that stent oversizing is a cause of occlusion [30]; hence, the diameter of the stent remains controversial. At our institution, anticoagulation was used in all cases to avoid obstruction as much as possible. However, Min et al. [32] were concerned about rebleeding after hemostasis and administered anticoagulation in only 35% of cases but found that the stent patency rate at 1 year was 52.9%, indicating that there was no association between the administration of anticoagulation and stent patency. Long-term tumor recurrence has been reported to cause stent failure [32]. However, we believe that collateral blood vessels will develop if the Viabahn stent graft is slowly occluded due to thrombus or other factors. Therefore, if bleeding from a GDA pseudoaneurysm can be promptly treated with a Viabahn stent, the incidence of stent failure is minimal.

This study had some limitations. First, it was retrospective because conducting a prospective study of cases with bleeding and life-threatening conditions is difficult. Second, because of the small sample size and the fact that the study was conducted at a single institution, the generalizability of the results may be limited. Replicating the results with larger sample sizes in future studies will provide more statistical power to evaluate the intervention’s efficacy.

## Conclusions

The Viabahn stent graft is useful for managing bleeding from a GDA pseudoaneurysm after PD. If hemostasis can be successfully controlled immediately, the possibility of stent failure is not a major concern.

### Electronic supplementary material

Below is the link to the electronic supplementary material.

Supplementary Material 1

## Data Availability

The datasets used and/or analysed during the current study available from the corresponding author on reasonable request.

## List of abbreviations

DP: distal pancreatectomy
PD: pancreatoduodenectomy
TAE: Transcatheter arterial embolization
TP: total pancreatectomyx
SSPPD: subtotal stomach-preserving PD
CHA: common hepatic artery
QVA: quantitative vessel angiography
PHA: proper hepatic artery
GDA: gastroduodenal artery
CTCAE: Common Terminology Criteria for Adverse Events
GIST: gastrointestinal stromal tumor
POPF: postoperative pancreatic fistula
CRP: C-reactive protein
